# Quality of Life, Neurosensory Disorders and Co-Occurring Medical Conditions in Individuals on the Spectrum, with a Special Focus on Females Diagnosed with Autism: A Systematic Review

**DOI:** 10.3390/jcm12030927

**Published:** 2023-01-25

**Authors:** Camron Davies, Moeed Moosa, Keelin McKenna, Jeenu Mittal, Idil Memis, Rahul Mittal, Adrien A. Eshraghi

**Affiliations:** 1 Hearing Research and Communication Disorders Laboratory, Department of Otolaryngology, Miller School of Medicine, University of Miami, Miami, FL 33136, USA; 2 Herbert Wertheim College of Medicine, Florida International University, Miami, FL 33199, USA; 3 Department of Neurological Surgery, Miller School of Medicine, University of Miami, Miami, FL 33136, USA; 4 Department of Pediatrics, Miller School of Medicine, University of Miami, Miami, FL 33136, USA

**Keywords:** autism spectrum disorder, women diagnosed with autism, quality of life, neurosensory disorders, psychiatric conditions

## Abstract

Autism Spectrum Disorder (ASD) is a neurodevelopmental disorder that has a high prevalence and a significant economic impact. Our knowledge regarding neurosensory disorders and co-occurring medical conditions in the ASD population is limited, particularly for autistic women. Most of the studies include male participants or do not make comparisons with their female counterparts. The objective of this systematic review article is to explore the quality of life as well as the prevalence of neurosensory disorders and co-occurring medical conditions in individuals on the spectrum, with a special focus on autistic females. The literature search was carried out in accordance with the Preferred Reporting Items for Systematic Reviews and Meta-Analyses (PRISMA) criteria. A protocol of this systematic review was designed a priori and was registered in the PROSPERO database (registration number: CRD42022330368). We concluded that numerous medical areas were of concern. Autistic females are more likely than their male counterparts with ASD to suffer from psychiatric conditions such as post-traumatic stress syndrome, depression, and eating disorders. They are also more likely to report GI-related disturbances and chronic pain. Further investigations are warranted to determine quality of life, as well as the prevalence and severity of neurosensory disorders in individuals with ASD, specifically studies comparing autistic females with their male counterparts. The information derived from these studies will help develop better support systems for individuals with autism, particularly females on the spectrum, in pursuit of improving their quality of life.

## 1. Introduction

### 1.1. Autism Spectrum Disorder Prevalence and Economic Burden

Autism Spectrum Disorder (ASD) is a neurodevelopmental condition with three core features: poor social interactions, communication challenges, and repetitive behaviors, interests, or activities [1]. In the United States, ASD’s current prevalence is 1 in 44 children, and this continues to increase [2]. ASD is a significant healthcare and social burden. In the United States, it is estimated that by 2025, ASD will cost the country USD 460.8 billion, which is 1.6% of the GDP [3]. This estimate was generated using criteria including the number of persons diagnosed with ASD, the money spent on both medical and non-medical care, and lost productivity from parents and individuals suffering from ASD [3]. If this estimate is correct, the burden will outweigh that of both diabetes and attention deficit hyperactivity disorder (ADHD) in the United States [3]. Cakira et al. also extrapolated estimates for the United States using measures including direct medical costs, special education, loss of productivity, and living support [4]. From 1990–2019, there were an additional 2 million diagnoses of ASD, introducing a social burden of USD 7 trillion. If this rate continues, an additional USD 4 trillion in cost is envisaged by 2029 in the United States [4].

### 1.2. Quality of Life

In addition to this high economic burden, adults diagnosed with ASD have a reduced quality of life (QoL) in comparison to their neurotypical counterparts [5]. QoL is often a difficult concept to define, but it is generally thought of as “the impact of perceived health on an individual’s ability to live a fulfilling life” [6]. QoL measures are important to understand, as they allow us to improve care for patients and to modify treatments appropriately [6]. QoL is a versatile factor that can be investigated in adults with ASD. However, there is limited research on how QoL is affected by sex or age [5].

### 1.3. Gender Differences

There are many apparent differences between men and women who are diagnosed with autism. ASD is more common in males compared to females, and in general, females are often diagnosed with ASD later in life than males [7]. Thus, gender is an important factor when diagnosing autism in adults. There have also been reports of women with ASD feeling conflicted between ASD-related characteristics and their traditional feminine identity [8,9]. Therefore, this could indicate a possible effect of gendered socio-cultured systems and expectations for well-being in women with ASD [7]. Women diagnosed with ASD are also at an increased risk of poor health outcomes and have higher healthcare costs compared to males [10,11]. Central sensitivity syndrome (CSS) is more commonly diagnosed in women, and they tend to have greater sensitivity to pain, and heightened sensitization compared to their male counterparts. Both men and women with ASD are vulnerable to anxiety and mood disorders, but women with ASD are at higher risk of suicidal behaviors.

### 1.4. Diagnostic Criteria

There are also vast differences between males and females with ASD and their criteria for diagnosis. When symptoms such as intellectual disability and behavioral difficulties are milder, females appear to be less likely to receive an ASD diagnosis than males. This suggests that females may need to show more characteristic symptoms than males to receive a diagnosis [12]. When looking at younger children with ASD, girls experience more difficulties than boys in terms of communication and sleep problems [13].

### 1.5. Objective

The objective of this systematic review is to discuss the QoL, neurosensory disorders, and co-occurring medical conditions in individuals diagnosed with ASD, with a particular focus on females with autism. Enhancing knowledge of such medical challenges encountered by individuals with ASD, especially women, will assist in the development of targeted interventions to effectively provide support to this population.

## 2. Materials and Methods

### 2.1. Search Strategy

This review was conducted in accordance with the Preferred Reporting Items for Systematic Reviews and Meta-Analyses (PRISMA) statement, with guidance from the Cochrane Collaboration Handbook [14,15,16,17]. A protocol of this systematic review was designed a priori and was registered in the PROSPERO database (registration number: CRD42022330368). Searches were performed in the PubMed, Science Direct, Web of Science, Scopus, and EMBASE databases [18] using the following MeSH terms: “co-morbidities in individuals with ASD”, “neurosensory disorders in individuals with ASD”, “Quality of life of adult individuals with ASD”, “stress in adult individuals with ASD”, “psychiatric disorders in individuals with ASD”, “women diagnosed with autism and comorbidity”, and “medical conditions in women diagnosed with autism”. 

### 2.2. Study Selection

Two reviewers independently reviewed all searched titles, abstracts, and full-text publications (C.D. and M.M.). Disagreements about inclusion and exclusion criteria were resolved by consensus among the reviewers or discussion with other researchers involved in this study. All studies with a diagnosis of ASD based on gold-standard criteria such as DSM, ADOS, or CARS score were included. 

Exclusion criteria included research that did not meet the above criteria, review papers, meta-analyses, abstracts only, conference proceedings, editorials/letters, and case reports, as well as outcomes not relevant to this study. Articles selected based on initial analysis of abstracts were further extracted for full-text analysis.

### 2.3. Quality Assessment

The Joanna Briggs Institute Critical Appraisal Tool for Cohort Studies was used to assess the studies’ quality and risk of bias [19,20,21]. This assessment was completed separately by two investigators (C.D. and M.M.), and any disagreements were managed by consensus among the reviewers or discussion with additional research investigators.

## 3. Results

### 3.1. Study Selection

**Figure 1:**
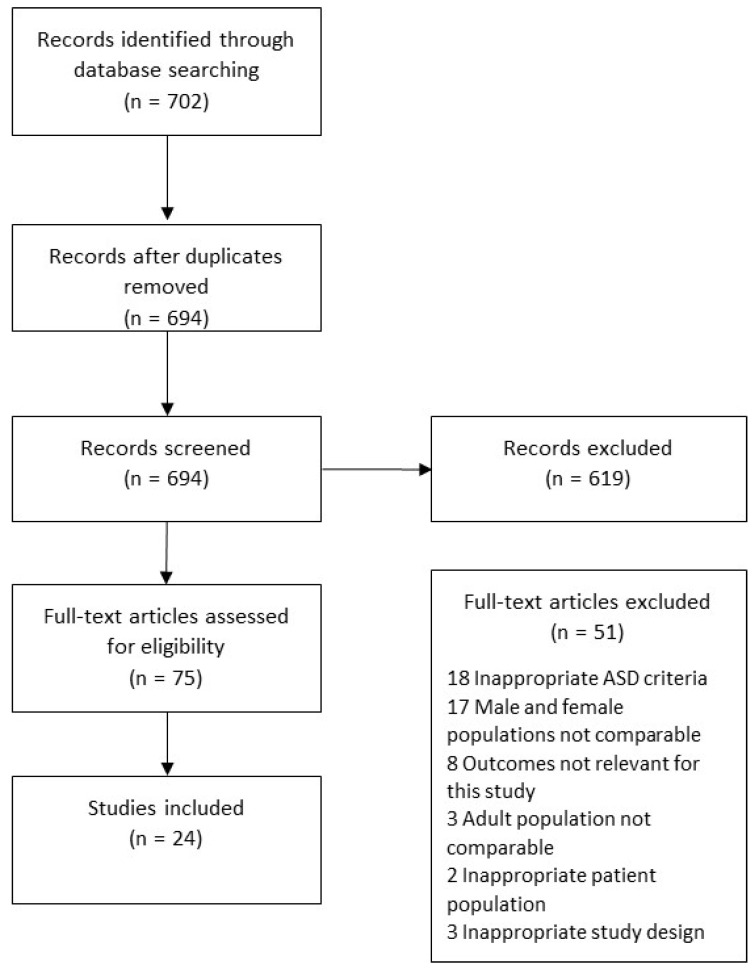
Flowchart of Preferred Reporting Items for Systematic Reviews and Meta-Analyses (PRISMA) detailing study.

A total of 702 articles were initially identified. Of these, 75 studies were selected after excluding duplicates or irrelevant articles. Following the full-text review, a total of 24 articles were selected for this study after excluding 51 studies for reasons outlined in the PRISMA diagram, such as outcomes not relevant to this study or ASD (Figure 1).

### 3.2. Quality Assessment of Included Studies

The critical review of the studies included in this systematic review is shown in Table 1.

### 3.3. Data Extraction and Study Outcomes

A summary of the main findings of studies included in this review article has been shown in Table 2.

#### Quality of Life

Three studies assessed the quality of life (QoL) of individuals with ASD. Asztély et al. conducted a longitudinal study which aimed to identify chronic pain in 100 Swedish females with ASD and/or ADHD since childhood or adolescence [22]. This study also determined the health-related quality of life (HRQoL) in this Swedish women’s cohort, as well as the probable relationship between chronic pain and HRQoL. The researchers discovered that 34 of these women had ASD as their primary diagnosis as children, while 31 had ADHD as a secondary diagnosis. It was observed that 76.6% of these women reported chronic pain, and women with an ADHD diagnosis prescribed ongoing stimulant treatments reported a lower prevalence of chronic widespread pain (CWP) (32.5%) compared to those without stimulant treatment. Within this group of 59 women with ASD and/or ADHD who reported chronic pain, 26% reported recurrent headaches, 29.9% experienced abdominal pain, while 14.3% reported both headaches and abdominal pain, underlining the comorbidities associated with chronic pain. Given these findings, including those women receiving a diagnosis of ASD and/or ADHD, there is a need to discuss pain problems during clinical visits.

A cross-sectional study conducted by Braden et al. was performed in two parts. The first part utilized a 36-item survey comparing mental and physical HRQoL scores in adults with ASD and neurotypical matched adults [5]. Mental HRQoL was shown to be worse in both males and females with autism compared to neurotypical individuals. However, physical HRQoL is only worse in women with ASD. They then performed the second part of the study; a randomized pilot evaluation approach was applied to assess the effect of mindfulness-based stress reduction (MBSR). When compared to individuals with ASD receiving support/education intervention, MBSR improved disability-related QoL to a greater extent than was experienced by those receiving support/education intervention alone. Furthermore, the combination of support/education with MBSR was more effective in improving HRQoL in women diagnosed with ASD. A novel conclusion from these studies is that older age is related with improved mental HRQoL, exclusively in women with ASD. For both studies, there were no significant baseline differences amongst participants. The first part of the study addressed several limitations. First, it was limited by underpowered interaction effects (70% power for diagnosis by sex interaction) and the inability to calculate power for the three-way diagnosis. The age relationships were also a limitation, as there could be possible cohort effects. Another limitation was that the ASD and neurotypical groups were not matched for psychiatric comorbidity conditions. Adults with ASD and comorbid intellectual disabilities (IQ < 70) were excluded, which limits the generalizability to the ASD community. The use of an active control group to determine the advantages of mindfulness while seeking to control for social support, stress reduction education, and bias was a strength of the second portion of the study. However, due to the exclusion criteria of adults with ASD and IQ scores < 70, they were unable to generalize the findings to a broader population of adults with ASD and examine the scope of intellectual abilities that can benefit from these interventions. Ultimately, these findings could be further investigated by using a larger cohort and employing precision medicine strategies to help improve the QoL of adults with ASD throughout their life [5].

A cross-sectional study by Leader et al. was performed on 107 adults with an ASD diagnosis. A questionnaire was used to examine the relationships between sleep problems, gastrointestinal (GI) symptoms, autism traits, social support, and social functioning and QoL [23]. They found that GI-related symptoms were a common comorbidity amongst 86% of the participants, and 89% of the individuals were regarded as poor sleepers. The more severe the sleep issues, the poorer the QoL became in physical health and environmental domains. Poorer QoL in the environmental domain was associated with daytime dysfunction and sleep duration. A statistically significant multiple regression model for regulating subjective sleep quality, sleep latency, sleep duration, habitual sleep efficiency, sleep disruptions, sleep medication usage, and daytime dysfunction was developed. (F_(7, 99)_ = 3.83, *p* = 0.001, R^2^ = 0.21, adjusted R^2^ = 0.16). They also discovered that higher levels of social support were associated with higher levels of QoL in the psychological, social, and environmental domains. Finally, they discovered that lower social functioning was associated with lower QoL across all four domains. The general model that controlled for social functioning was statistically significant as well (F_(1, 105)_ = 64.37, *p* < 0.001, R^2^ = 0.38, adjusted R^2^ = 0.37). A limitation of this study was the use of a self-report questionnaire dependent on subjective accounts. Another limitation was that causality could not be inferred, as this was a cross-sectional study [23].

### 3.4. Neurosensory Disorders

In a study by Grant et al., in the Netherlands, 973 autistic people (410 men and 563 women) completed questionnaires on autistic characteristics, sensory sensitivity, central sensitivity syndrome (CSS), and physical and mental health problems. The purpose of this study was to investigate the prevalence of CSS diagnoses and symptoms in autistic adults, as well as to see if CSS symptoms were connected to autism attributes, mental health, sensory sensitivity, or gender [24]. The researchers found that 208 participants (21.4%) reported one or more CSS diagnoses (*p* < 0.001) and 582 individuals (59.8%) met or exceeded the clinical cut-off for CSS. They also found CSS women were more likely to report a CSS diagnosis and experienced more CSS symptoms than men. CSS symptoms had the following predictors that were significant: sensory sensitivity, anxiety, age, and gender. Sensory sensitivity and anxiety were discovered to be mediators between autistic features and CSS symptoms. A strength of this research study was that since the participants were not directly told the aims of the study, these data were reported as an ongoing data collection. Therefore, the odds of selection or attrition bias were lower; thus, these findings are likely not inflated. A major limitation of this study was the lack of control group; thus, no major statistical power could be calculated. The sample population was mostly Dutch, making it less generalizable to other ethnicities. Another limitation was the gender variable was assessed by asking participants to check a box. This allows individuals to perceive this as either gender assigned at birth or gender of identification. Lastly, another limitation to consider was the wording of the Central Sensitization Inventory (CSI), as it can imply different associations between the overlap of traits [24].

In another study by Boogert et al., 101 Dutch participants with ASD completed three types of questionnaires regarding sensory processing and aggression. The goal of this study was to determine whether there was a direct link between sensory processing and violent behavior in individuals with ASD [25]. The researchers found that difficulties in sensory processing were associated with more aggressive behavior (*f*^2^ = 0.25), more proactive (*f*^2^ = 0.19) and reactive aggression (*f*^2^ = 0.27), more physical aggression (*f*^2^ = 0.08), more verbal aggression (*f*^2^ = 0.13), more anger (*f*^2^ = 0.20), and more hostility (*f*^2^ = 0.12). They also discovered a link between the neurological threshold and the behavioral response to aggression and hostility. Furthermore, those who scored higher in sensory sensitivity than the control group had a higher likelihood of aggressive conduct. Out of the 19 participants with the highest total aggression scores, 15 had elevated scores on low registration (*p* = 0.071). All 19 participants had elevated scores for sensory sensitivity (*p* = 0.001). There were no statistically significant variations in the degree or direction of the correlations between sensory processing and aggressive behavior. Some limitations of this study were that even though differences were demonstrated in sensory processing variables, the confounding effects of gender were limited. Lastly, there was no implication that the findings could be generalized to other clinically relevant populations [25].

### 3.5. Neurological and Psychiatric Disorders

Of the studies included in the systematic review, nine investigated the co-presence of various psychiatric disorders in individuals diagnosed with ASD. Barnard-Brak et al. examined the death records of 1754 people who had ASD indicated as one of the reasons for death. The frequency data for these deaths were obtained and a weighting variable was created to adjust for the variability of the prevalence rates and alterations of the ASD diagnostic criteria. Analyses of these data suggest that females diagnosed with ASD had a higher mean age at death compared to males diagnosed with ASD [26]. These findings were consistent with the general population’s trend in sex differences. Furthermore, those with autism were less likely than the general population to get Alzheimer’s disease or a form of dementia. When assessing males and females with ASD, males were more likely to have acquired ASD or a form of dementia compared with females. A limitation of this study was the prevalence rates before 1992 were difficult to obtain. Another limitation was they only selected for individuals who identify with ASD in general and not based on any other characteristics. The results of this study showed that individuals with ASD do not have a low survival rate, and thus need services that help support their lives [26].

Bishop et al. investigated the prevalence, incidence, and antiepileptic drug use associated with epilepsy in 21+ aged autistic adults with (N = 2738) and without (N = 4775) intellectual disability in the United States using population-level Medicaid data from the Wisconsin Department of Health Services Medicaid program [27]. They also compared these results with 18,429 adults with intellectual disability only and no ASD. In comparison to 27% of people with intellectual impairment, 34.6% of autistic adults with intellectual disability and 11.1% of autistic adults without intellectual disability had epilepsy. In autistic individuals, female sex and intellectual impairment were similarly linked to an increased incidence of seizures. The prevalence of epilepsy in autistic women in this population cohort was 1.2 times that of autistic men. The lack of a connection between age and epilepsy prevalence in autistic individuals without intellectual impairment was one of the study’s limitations. This might be attributable to cohort effects, since older autistic adults with intellectual impairment vary from younger autistic adults with intellectual disability in terms of age and health-related impacts [27].

In another study by Haruvi-Lamdan et al., 25 ASD and 25 neurotypical individuals between the ages of 18 and 35 answered questionnaires on potential traumatic life experiences of a social or non-social nature, as well as autistic features [28]. These participants were similar in age to one another, and the male to female ratios were comparable between the groups. This study intended to investigate the relationship between traumatic life experiences and PTSD symptoms. They discovered a higher prevalence of likely PTSD in the ASD group (32% vs. 4% in the neurotypical adult group), and those with ASD reported greater PTSD symptoms, as well as re-experiencing PTSD symptoms (PTSS) and hyper-arousal. Re-experiencing symptoms include recurring and disturbing experiences, nightmares, or flashbacks. Females with ASD also reported more negative life events than neurotypical females. In addition, 60% of participants with ASD chose a social event as their most distressful event compared to 20% of typical adults. They also found that individuals with ASD and probable PTSD co-occurrence resulted in poorer social skills versus just ASD alone. Since this study used a small sample size, it was limited due to its focus on adults with ASD and no apparent intellectual or verbal impairments. Thus, it was difficult to generalize these findings to a larger ASD population. The study also had a lack of statistical power due to their sample size. These results showed that individuals with ASD have increased vulnerability to traumatic experiences and PTSD, particularly due to social stressors. Furthermore, females may be more vulnerable to PTSD [28].

Jones et al. conducted a 25-year outcome study on individuals diagnosed with autism in Utah in the United States. This study included 92 child participants selected during the 1980s. Eleven participants died during the 25-year span of the study. The study subjects were given questionnaires regarding their medical symptoms, disorders, hospitalizations, surgeries, and use of medication [29]. Females with ASD showed increased medical comorbidities (*p* = 0.01), but not increased prevalence of intellectual disability (*p* = 0.79). The researchers also found that these adults diagnosed with ASD experienced a greater incidence of medical conditions such as obesity, insomnia, and seizures, despite their intellectual ability. There was no statistically significant difference between the participants in the medical comorbidities follow-up groups and their intellectual functioning. There was also no significant difference between the frequency of psychotropic medication use amongst individuals with autism with and without hypertension, hyperlipidemia, or diabetes, as well as based on Body Mass Index (BMI). A limitation of this study was participants were limited to adults whose ASD was ascertained during childhood according to social development concerns in the 1980s, and as a result, it is possible that high-functioning individuals with ASD were not included in the study population. This study also had low statistical power for detecting a difference between intellectual disability or severity in the groups of medical conditions. Lastly, due to the nature of the questionnaires given to the participants, another restriction was the lack of medical record information to offer collateral medical history [29].

Zheng et al. collected information on 315 young adults with a childhood diagnosis of autism for current depressive symptoms, depression diagnosis, and treatment status. They determined that 65.4% of these young individuals had depression and 46.7% of them reached the clinical cut-off for depression based on depressive symptom assessments [30]. Females were more likely to receive a formal depression diagnosis versus males (*p* = 0.01). At the time of the study, 91% of depressive females and 74.7% of depressed men had ever been diagnosed with depression. Furthermore, race was not a strong predictor of depression diagnosis (*p* = 0.50). The form of treatment for depression was medication, followed by therapy. A total of 58.5% of the depressed people in the research were receiving therapy, and 68% had previously received treatment. It was also shown that people with a formal diagnosis of depression and greater levels of education had a higher chance of receiving treatment for depression. Possible reliability concerns with the self-reported depression diagnosis and the lack of matching criteria when comparing the cut-offs on the depressive symptom measures to the criteria for a clinical diagnosis of depression were some of the study’s shortcomings. This study was also confined to people with a childhood autism diagnosis; therefore, it may not be generalizable towards certain adult populations. Furthermore, a large portion of the sample (80%) was white, and thus it was difficult to detect meaningful effects of race based on that sample; a more diverse sample would have been needed. A strength of this study was the ability to assess for various types of depression experiences, given that a relatively large number of participants were included. This study also used a community sample from a national autism research registry, which addressed some of the sampling biases that might occur with individuals suffering from mental health concerns [30].

Murray et al. investigated the symptom profiles of anxiety and depression of 205 adults referred to the Autism Diagnostic and Research Centre (ADRC) between 2011 and 2015 for a confirmed ASD diagnosis. They discovered that 37% of the population had moderate or severe anxiety, and 46% had moderate or severe depression [31]. They also discovered that anxiety was connected with younger age and greater self-reported autistic symptom severity and was higher in females. There was a statistically significant difference between females who reported more anxiety-like symptoms compared to males (*p* = 0.052). A limitation of this study was that it was not longitudinal, and thus the direction of its effects was unable to be followed. Additionally, only the current self-reported trait symptoms of anxiety and depression were considered, rather than a comprehensive clinical diagnosis or a longitudinal lifetime pattern experience. Lastly, only trait measures of anxiety and depression were investigated, not other psychiatric disorders [31].

**Table 1:** Assessment of risk of bias of the included studies.

Lead Author and Date	1 ^a^	2 ^a^	3 ^a^	4 ^a^	5 ^a^	6 ^a^	7 ^a^	8 ^a^	9 ^a^	10 ^a^	11 ^a^
Asztely et al. [22], 2019	N	Y	Y	N	N	Y	Y	Y	Y	N	Y
Barnard-Brak et al. [26], 2019	N	Y	Y	Y	N	Y	N	Y	Y	N	Y
Bishop et al. [27], 2021	Y	Y	Y	N	N	N	Y	N	N	N	Y
Braden et al. [5], 2022	N	Y	Y	Y	Y	U	Y	Y	Y	Y	Y
Charman et al. [32], 2017	Y	Y	Y	N	N	U	Y	Y	Y	Y	Y
Darbro et al. [33], 2016	Y	Y	Y	N	N	U	Y	NA	NA	N	Y
DaWalt et al. [10], 2021	Y	Y	Y	Y	Y	NA	Y	Y	Y	Y	Y
Gesi et al. [34], 2021	Y	Y	Y	U	U	U	Y	Y	Y	U	Y
Grant et al. [24], 2022	Y	Y	Y	Y	Y	Y	Y	Y	U	U	Y
Haruvi-Lamdan et al. [28], 2020	Y	Y	Y	Y	Y	Y	Y	Y	N	U	Y
Hirvikoski et al. [9], 2016	Y	Y	Y	Y	Y	Y	Y	Y	Y	Y	Y
Jones et al. [29], 2015	N	Y	Y	Y	N	Y	N	Y	N	N	Y
Lai et al. [35], 2019	U	Y	Y	Y	U	Y	Y	U	U	U	Y
Leader et al. [23], 2021	N	Y	Y	Y	Y	Y	N	NA	NA	NA	Y
McGillivray et al. [36], 2014	Y	Y	Y	Y	Y	U	Y	Y	Y	U	Y
Miesen et al. [37], 2018	N	Y	Y	N	N	U	Y	Y	N	N	Y
Murray et al. [31], 2018	Y	Y	Y	Y	Y	U	Y	Y	U	Y	Y
Power et al. [38], 2013	Y	Y	Y	Y	Y	U	Y	Y	U	Y	Y
Rodgaard et al. [39], 2021	Y	Y	Y	N	N	Y	Y	Y	Y	NA	Y
Spek et al. [40], 2019	Y	Y	Y	Y	Y	Y	Y	Y	Y	Y	Y
van den Boogert et al. [25], 2021	Y	Y	Y	Y	Y	U	Y	Y	Y	Y	Y
van der Linden et al. [41], 2021	N	Y	Y	N	Y	NA	Y	NA	NA	NA	Y
Weir et al. [42], 2021	Y	Y	Y	Y	Y	Y	Y	Y	Y	Y	Y
Zheng et al. [30], 2021	N	N	Y	Y	Y	Y	N	Y	U	NA	Y

^a^: The numbers correspond to the quality assessment criteria as described in the table below: 1. Were the two groups similar and recruited from the same population? 2. Were the exposures measured similarly to assign people to both exposed and unexposed groups? 3. Was the exposure measured in a valid and reliable way? 4. Were confounding factors identified? 5. Were strategies to deal with confounding factors stated? 6. Were the groups/participants free of the outcome at the start of the study (or at the moment of exposure)? 7. Were the outcomes measured in a valid and reliable way? 8. Was the follow-up time reported and sufficient to be long enough for outcomes to occur? 9. Was follow up complete, and if not, were the reasons to loss to follow up described and explored? 10. Were strategies to address incomplete follow-up utilized? 11. Was appropriate statistical analysis used? Y—Yes (green color); N—No (red color); U—Unclear (orange color); NA—Not applicable (orange color)

**Table 2:** A summary of main findings of included studies.

Reference	Study	Population	Exposure	Comparison	Outcomes
Asztely et al. [22], 2019	Single arm cohort study	77 females aged 19 through 37 years in Gothenburg, Sweden.	ASD and/or ADHD	Females with ASD +/− ADHD vs. Females with ADHD	- Increased chronic widespread pain (CWP) - Increased NCP in individuals with ASD - No difference in reported number of painful regions - No statistically significant differences with regard to abdominal pains or headache - Decreased HRQoL
Barnard-Brak et al. [26], 2019	Non-randomized trial	1754 individuals with ASD	ASD, Alzheimer’s Disease, and dementia	Average age at death for males with ASD vs. females with ASD Males diagnosed with ASD with dementia vs. females diagnosed with ASD with a dementia	- Increased age at death in females - Dementia less likely to be a cause of death - Males more likely to have Alzheimer’s disease/dementia
Bishop et al. [27], 2021	Non-randomized trial	4775 ASD without ID 2738 ASD with ID 18,429 with ID without ASD	ASD, epilepsy, and ID	Males with ASD and ID and females vs. Males with ASD but without ID and females vs. Males with ID but without ASD and females	- Increased risk of epilepsy captured in a shorter time period - Increased epilepsy in ID compared to ASD - Epilepsy prevalence was impacted by age - Increased prevalence of epilepsy in women with ASD compared to men
Braden et al. [5], 2022	Cross sectional study	Study 1: 67 participants with ASD and 66 Neurotypical (NT) participants. Study 2: 70 participants	Modified MBSR	Males with ASD vs. Females with ASD NT Males vs. NT Females	Study 1: - No significant differences between ASD and NT participants on age, IQ, or sex distribution- Physical HRQoL decreased in women with ASD Study 2: - No significant differences between MBSR and support/education participants on age, IQ, sex distribution, or ASD severity - Women improved more than men in BREF physical and psychological scores
Charman et al. [32], 2017	Randomized controlled trial	737 participants	ASD	Males with ASD vs. Females with ASD	- No sex differences on parent-report questionnaire - No age difference on ADOS and ADI-R - Decreased symptom severity in adults with ASD on SRS-2 - Increased symptoms on SRS-2 in ASBQ and AQ adolescent females compared to males
Darbro et al. [33], 2016	Case-control study	1837 patients with ASD	ASD	Patients with ASD vs. patients without ASD	- Increased coding variation in oncogenes in individuals with ASD - Decreased rates of cancer in individuals with ASD
Gesi et al. [34], 2021	Observational Cross-Sectional Study	61 adult patients with ASD without language or intellectual disability	ASD	Males with ASD vs. Females with ASD	- Increased age at diagnosis in females - Women’s age at diagnosis of ASD positively correlated with the AdAS Spectrum Verbal communication and restricted interests and rumination scores - Males more likely to be diagnosed with ASD
Grant et al. [24], 2022	Cross-sectional study	973 adults with autism	ASD and CSS	Males with ASD vs. Females with ASD	- CSS symptoms common in people with ASD - Increased pain and fatigue in individuals with ASD - Higher scores on the CSI associated with greater sensory sensitivity, greater anxiety, and lower subjective well-being. - Increased CSS diagnosis and severity in women - Greater sensory sensitivity, anxiety, and depression in women
Haruvi-Lamdan et al. [28], 2020	Cross-sectional study	Fifty adults aged 18–35	ASD and PTSS	Adults with ASD vs. Typical adults Females with ASD vs. Typical females Females with ASD vs. Males with ASD	- Higher levels of PTSS in ASD participants, especially females - Increased deficit in social skills when ASD/PTSD are combined
Hirvikoski et al. [9], 2016	Cohort study	54,168 individuals with ASD	ASD +/− ID +/− ADHD	Males with ASD vs. Females with ASD Females with ASD vs. Neurotypical Females	- Increased suicidal behavior/attempts in females compared to males
Jones et al. [29], 2015	Cohort study	92 males and females	ASD	Males with ASD vs. Females with ASD	- Increased prevalence of comorbid conditions in females
Lai et al. [35], 2019	Non-randomized trial	Typically developing males/femalesAutistic males/females	Autism and neurotypical behavior	Males with ASD vs. Females with ASD Females with ASD vs. Neurotypical Females	- Hypoactive right temporoparietal junction mentalizing and ventromedial prefrontal cortex self-representation responses in men with autism - Heightened ventromedial prefrontal cortex self-representation response with increased camouflaging in women with ASD - Lack of impaired neural self-representation and mentalizing in women with ASD
Leader et al. [23], 2021	Cohort study	107 females and males	ASD	Males diagnosed with ASD vs. Females diagnosed with ASD	- The effect of SFQ total on Physical Health Domain QoL was more negative for female participants - The effect of PSQI Total on Physical Health Domain QoL was more negative for male participants - The effect of SFQ total on Psychological Health Domain QoL was more negative for female participants - The effect of MSPSS Total on Social Relationship Domain QoL was more positive for male participants - The effect of SFQ total on Environment Domain QoL was more negative for female participants - The effect of PSQI Total on Environment Domain QoL was more negative for male participants
McGillivray et al. [36], 2014	Non-randomized trial	109 males and females	Asperger's Syndrome and Autism	Males with ASD vs. Females with ASD Females with ASD vs. Neurotypical Females	- Increased stress during everyday life events in female participants - Higher scores on the DASS anxiety subscales in females and males with ASD - Higher scores on the DASS stress subscales in females and males with ASD - Increased age correlated with increased stress in everyday life events in participants with ASD - Higher scores on sensory/personal contact, pleasant events, and social and environmental interaction SSS subscales in females
Miesen et al. [37], 2018	Cross-sectional study	807 adults	ASD	Males with ASD vs. Females with ASD Females with ASD vs. NT Females	- Males and females with ASD more likely to endorse transgender feelings
Murray et al. [31], 2018	Non-randomized trial	Adults with and without ASD	ASD	Males with ASD vs. Females with ASD in ASD, anxiety, and depression	- Increased moderate/severe anxiety and depression in females - Positive correlation between total anxiety and total depression scores - Decreased symptoms of anxiety with aging
Power et al. [38], 2013	Non-randomized trial	Male and Female Adults	Schizophrenia, depression, autism, substance abuse, and bipolar disorder	ASD and other Psychiatric Disorders Males vs. Females	- Individuals with autism had significantly fewer children - Across disorders, affected men had a consistently greater reduction in fecundity than affected women
Rodgaard et al. [39], 2021	Cross-sectional study	2199 males and females	ASD diagnosis after age 18	Males with ASD vs. Females with ASD	- No effect between sex and adult autism diagnosis - The majority of males and females had not been diagnosed by the age of 18 - Only 16% of males and 9% of females had received any of the diagnoses before the age of 12
Spek et al. [40], 2019	Non-randomized trial	Males and females with ASD, with and without housing and residential support	ASD	Males with ASD vs. Females with ASDFemales with ASD vs. Neurotypical Female Men with ASD and housing/residential support vs. men with ASD without support Men without psychiatric diagnosis	- Males with ASD showed difficulty adapting their eating behaviors for others - Males with ASD showed difficulty multitasking during meals - Women with ASD showed increased scores in perception, mealtime surroundings, social situation at mealtime, and simultaneous capacity - Women with ASD showed increased scores purchase of food and eating behavior
van den Boogert et al. [25], 2021	Observational Study	Males and Females with ASD	ASD	Males with ASD vs. Females with ASD	- Increased aggressive behavior with increased sensory processing difficulty - Adults with ASD who had higher scores in sensory sensitivity had the highest risk of aggressive behavior
van der Linden et al. [41], 2021	Cohort study	Male and Female Adults	ASD	Males with ASD vs. Females with ASD	- Male/female sex had no moderating effect on either emotional or biological stress reactivity
Weir et al. [42], 2021	Survey	Adults with and without ASD	ASD.	Males with ASD vs. Females with ASD Females with ASD vs. Neurotypical Females	- Differences in physical health comorbidity across all three models, regardless of both demographic and lifestyle-related factors - Increased risk of developing respiratory conditions, asthma, cardiovascular conditions, and prediabetes in autistic women - Increased risk of cardiovascular conditions, high cholesterol, arrhythmias, and Type II diabetes in autistic males
Wise et al. [43], 2017	Cross sectional study	Male and Female adults	ASD	Males with ASD vs. Females with ASD	- Females more likely to engage in screaming and oppositional behavior
Zheng et al. [30], 2021	Cohort study	Male and Female Adults	ASD	Males with ASD vs. Females with ASD	- Females more likely than males to receive a formal depression diagnosis - Race not associated with likelihood of diagnosis

In another study, Charman et al. recruited 437 children with ASD, adults with ASD, and 300 controls between 6 and 30 years of age. They conducted an in-depth clinical characterization of the EU-AIMS Longitudinal European Autism Project (LEAP) including observational, interview, and questionnaire measures of ASD phenotype [32]. They also assessed age, gender, and IQ differences in ASD core symptoms and common co-occurring psychiatric disorders, specifically anxiety, depression, and ADHD, using the DSM rating scale. Adults had more severe ASD symptoms than adolescents, and adult females reported more severe symptoms than men. There was a moderate association between higher ASD symptom scores and lower IQ. A limitation of this study was that due to the limited availability of the self-reported data, only parent-reported levels of ADHD symptoms were analyzed. Another limitation was that ASD participants with severe intellectual disability were excluded [32].

In the next study, Wise et al. conducted a naturalistic assessment of 74 people aged 30 and up who fit the DSM-5 criteria for ASD. The primary goal of this study was to investigate the long-term trajectory of behavioral and neuropsychiatric symptoms (BNPS) in 114 individuals with ASD who attended the Community Services for Autistic Adults and Children organization between 1981 and 2009 [43]. The secondary goal was to identify changes in comorbidities, BNPS, intellectual impairment, and linguistic abilities across younger and older cohorts within the sample. They discovered that gastrointestinal (68.9%) and epileptic disorders (23%) were frequent, and that 25.7% of the individuals had a BMI greater than 30. Females were also shown to be more likely to participate in screaming (*p* < 0.05) and oppositional conduct (*p* < 0.05). There was no statistically significant influence on BNPS prevalence. Among 39 participants with full-scale IQ scores, there was no correlation between age and intellectual impairment. A strength of the study was the inclusion of a broad range of ages in the sample size. The data were also available for three decades, allowing for a longitudinal evaluation of the BNPS. One major limitation of this study was that participants were chosen from a particular location based on their need for extensive living and occupational assistance. This makes the results hard to generalize to the broader ASD population. Other limitations include retrospective data, cross-sectional descriptive data, and chart review for determining a medical condition [43].

Finally, Spek et al. looked at 53 males and 36 females with ASD who presented with eating disorders. The researchers also considered whether or not these participants had housing and residential support [40]. They were trying to assess whether adults with ASD with housing and residential support experience fewer eating problems than adults with ASD without support. These individuals with autism were compared with neurotypical men (30) and women (38). The researchers’ findings indicated men with ASD and particularly women with ASD experience vast eating problems. Women with ASD were also able to recognize symptoms of an eating disorder. Furthermore, women with ASD had considerably higher scores on Perception and Purchase of food, but men with ASD had significantly higher scores on the subscale Motor control than women with ASD. The results of this study are limited in their ability to be generalized to populations at large due to the small sample size and because the sample included adults with ASD with average or higher cognitive abilities. In addition, there could have been bias in the results, as the individuals with autism with housing and residential support were not diagnosed with standardized instruments; thus, these individuals could have had another neurodevelopmental disorder in addition to ASD. Lastly, the researchers did not inquire about medication use with the participants and therefore could not consider the role of certain medications that could potentially affect eating disorders [40].

### 3.6. Stress

Two studies assessed stress in individuals diagnosed with ASD. Lindin et al. conducted the first study, which employed an Experience Sampling Method to assess three forms of daily life stress (activity-related, event-related, and social stress), negative affect (NA), and cortisol levels in 50 adults with ASD and 51 controls. They found significant interactions between these groups and stress in the NA model. There were higher levels of NA (*p* < 0.001), activity-related (*p* < 0.001), event-related (*p* = 0.028), and social stress (*p* < 0.001) [41]. This shows that the ASD group reacted more substantially to emotional stress than the control group (*p* = 0.760). There were no significant interactions between groups and stress in the cortisol model. As a result, individuals with ASD are more sensitive to emotional stress in reaction to unpleasant everyday life events and activities. This study’s strength was that it was the first electronic self-monitoring investigation on brief emotional and biological stress reactivity in individuals with ASD in the natural flow of everyday life; most of the participants had positive feedback regarding how to use the app and while filling out the daily questionnaires. Another strength was that multiple stressors were studied, since the study included individuals using medication and with comorbid disorders. A limitation was that there were some contrasting findings regarding ASD social stress, cortisol levels, and social motivation. Furthermore, because the stressors, cortisol, and NA were all measured at the same time, no causality could be deduced from the data [41].

In the second stress-related study by McGillivray et al., 109 individuals with ASD completed the Depression Anxiety Stress Scales (DASS) and the Stress Survey Schedule (SSS). The researchers were exploring gender and age variations in the frequency and severity of depression, anxiety, and stress symptoms as evaluated by the DASS and SSS subscales [36]. They were also attempting to compare the individuals’ levels of emotional distress with Australian normative data for the DASS subscales. They found that when comparing DASS depression, anxiety, and stress to Australian norms, males and females with autism showed elevated scores. Females aged 25–44 had significantly greater DASS depression subscale scores than males of the same age, as well as younger males and females. On the SSS subscales, there were substantial gender and age disparities. Females tended to show more stress than males during pleasant events, sensory/personal contact, and social and environmental subscales. They also found adult females to be more stressed regarding items related to Change, Social Threats, and Anticipation/Uncertainty. There were a few limitations for this study. The first was that the evaluation tools did not allow for a clinical diagnosis of depression or anxiety. The researchers also did not gather prior data on comorbidity diagnosis. Finally, the researchers were unable to establish the degree of autism symptomatology, since they did not measure or verify ASD features in the sample [36].

### 3.7. Other Comorbidities Associated with ASD

A study by DaWalt et al. analyzed the diagnostic codes of electronic health records from a healthcare system providing comprehensive care to more than 300,000 individuals diagnosed with ASD and compared the age and sex-matched controls. The researchers’ hypothesis was that autistic women have poorer health outcomes compared to autistic men and compared to women that are not autistic [10]. They discovered evidence that there was a multiplicative risk for certain domains, such as nutrition conditions and sleep disorders, and thus women with ASD faced double jeopardy by having higher rates of healthcare utilization within a domain than would be expected based on being female or having ASD alone. There was an additive risk for areas such as endocrine and gastrointestinal problems, which suggested that being both female and having ASD were related to increased healthcare consumption. There were no meaningful interactions discovered. This study had a large sample size from a rural area; however, the racial diversity of the patient population was limited. The researchers were also limited in the range of questions they could ask, and thus they were unable to account for variation in the practices of coding for billing [10].

An Italian observational cross-sectional study conducted by Gesi et al. evaluated certain features that were associated with misdiagnoses in 22 females and 39 males with ASD. These individuals had no language or intellectual deficits and were enrolled in the ASST Fatebenefratelli-Sacco in Milan for treatment of psychiatric comorbidities in adults with ASD [34]. The researchers gathered clinical history and two self-report questionnaires were given. The purposes of this research study were to: (1) assess autism spectrum symptoms in referred males and females; (2) assess sex differences in diagnostic delay and misdiagnosis rates; (3) determine whether certain domains of ASD symptomatology are associated with a greater diagnostic delay or the likelihood of misdiagnosis in women with ASD and men with ASD. They discovered that 75.4% of people were diagnosed with ASD an average of 8 years after their first examination by mental health professionals. Females had a much longer delay in re-referring to mental health services and were diagnosed with ASD at a significantly older age. Diagnostic delay was shown to be inversely associated with Adult Autism Subthreshold (AdAS) Spectrum overall scores, as well as verbal communication, empathy, inflexibility, and adherence to routine domains in males. This diagnostic delay was positively connected with AdAS Spectrum verbal communication, limited interests, and rumination domain scores in females. Among misdiagnosed participants, females outperformed men in the hyper/hypo-reactivity to sensory input domain. Females were also less likely than men to be accurately diagnosed and more likely to be misdiagnosed during the initial examination. This study was limited due to its small sample size and smaller percentage of females versus males; thus, proper statistical power was unable to be calculated. Another limitation was the sample population represented a limited population and thus did not allow for a broader ability to be generalized. Additionally, recall bias could have affected the findings, since part of the data collection was dependent on patient and relative reports. In contrast, this study lays the groundwork for future research into gender-specific information on ASD individuals seeking treatment for comorbid disorders [34].

In another study by Lai et al., a total of 119 individuals with ASD completed a task based on a functional magnetic resonance imaging (fMRI) paradigm to examine brain activity in the right temporoparietal junction and ventromedial prefrontal cortex during mentalizing and self-representation. The researchers investigated whether aberrant brain responses during mentalization and self-representation in autistic people are sex/gender dependent [35]. They also investigated whether camouflage is linked to sex/gender-specific brain responses. Camouflaging in autism was measured by comparing extrinsic behavior in social–interpersonal situations to intrinsic status. When compared to ordinarily developed males, autistic men have hypoactive right temporoparietal junction mentalizing and ventromedial prefrontal brain responses. However, the reactions of autistic women were no different from those of ordinarily functioning women. Only increased camouflage was linked to an increase in ventromedial prefrontal cortex self-representation. There were a few limitations of this study. The sample size was the largest reported to date; however, it was underpowered to detect small effect sizes. A larger sample size would be needed to validate the findings of this study. Another limitation was also due to the sample size, as this limited the capability to use a hypothesis-free discovery approach, since more statistical comparisons lead to larger statistical power declines. Furthermore, unresolved challenges with ascertainment and clinical evaluation impede research on sex/gender variations in autism. Another disadvantage was that there were no follow-up data about various life stages and the roles of experimental effects and sex/gender-related plasticity in the development of the social brain, since the age range was so vast. Finally, it can be difficult to distinguish sex effects from gender effects and hence connect the amount by which camouflage is associated with psychological dimensions [35].

Power et al. conducted research in which they assessed and compared the fecundity of individuals with schizophrenia, autism, bipolar illness, depression, anorexia nervosa, or substance misuse to that of the general population [38]. The primary goal of this study was to compare the reproductive fitness of patients with schizophrenia and other psychiatric illnesses to that of unaffected controls in order to assess the extent of selection on causative genetic variations. The outcomes of this study were that affected patients had significantly fewer children (*p* < 0.01), and this was consistently more applicable to men than women. Therefore, this suggests that male fertility and reproductive fitness is sensitive. Brothers of individuals with schizophrenia and autism had lower fecundity (*p* < 0.001). Furthermore, siblings of individuals suffering from depression and substance addiction had considerably higher fecundity (*p* < 0.01). The researchers also discovered that the sisters of patients with schizophrenia and bipolar illness had greater fecundity (*p* < 0.01), although this was a tiny sample of the population and not enough to compensate for the affected individuals’ reduced fitness. Therefore, there is a compelling suggestion that strong selection exists against schizophrenia, autism, and anorexia nervosa. A limitation of this study was that it relied on the assumption that the fecundity measured was an accurate reflection of the individual’s reproductive fitness [38].

A Danish study by Rødgaard et al. compared adults with autism (N = 2199) to a control population with no records of autism diagnosis (N = 460,798). The researchers counted the number of people who had received various psychiatric or neurological diagnoses as children. The primary aim of the study was to examine the diagnoses given in childhood among individuals who had an autism diagnosis in adulthood [39]. This was conducted to determine whether the late autism diagnosis might be explained by a childhood misdiagnosis or diagnostic overshadowing. They discovered that most childhood diagnoses were overrepresented in adults with autism. Furthermore, the most common childhood problems in this group were ADHD, affective disorders, anxiety, and stress disorders. The majority of childhood diagnoses were made after the age of 12. However, 69% of men and 61% of females with adult autism diagnoses did not acquire any researched diagnosis before the age of 18. A limitation of this study was that there could be data that are not completely included in the dataset from private practices, since moderate cases of psychiatric conditions are treated largely by family physicians or school psychologists. There is also the possibility for diagnoses to have insufficient validity, leading to false positives. Lastly, since the data were based solely on a Danish population, they are less generalizable to other populations [39].

In another study by Miesen et al., gender dysphoria (GD) was explored among individuals with ASD. This study compared 573 autistic adolescents (469 assigned boys and 104 assigned girls) and 807 adults (616 assigned males and 191 assigned females) with ASD to 1016 adolescents and 846 adults from the general population [37]. They measured emotional and behavioral problems with DSM-oriented scales. The adolescents and adults were also given social behavior questionnaires to measure specific subdomains of the ASD spectrum. When compared to the general population (3–5%), the researchers found that there were considerably more adolescents (6.5%) and adults (11.4%) with ASD. Adolescent girls with autism endorsed this more than boys with autism. There were no significant gender differences among adults with ASD. Furthermore, adolescents with autism and adults with autism who approved the gender item scored much higher than those who did not. There were no significant relationships between gender item endorsement and any specific ASD subdomain. However, there were several limitations of this study. First, a major limitation of this study was the lack of use of a control group or referred control groups. Second, utilizing only one item from each of the Youth Self-Report (YSR) and Adult Self-Report (ASR) was a limited measure of GD that did not account for non-binary gender identities. Lastly, the reliability of the self-report method might not be consistent in individuals with ASD versus normally developing individuals [37].

Weir et al. performed an online physical health survey on 2368 people, 1156 of whom were autistic. The major goal of this study was to look at the prevalence of cancer, cardiovascular disease, respiratory disease, and diabetes [42]. According to the findings of this study, autistic females are more likely than non-autistic men to have cardiovascular diseases, respiratory disorders, asthma, low blood pressure, and arrhythmias. Based on this, people with autism have a higher chance of developing these disorders than the general population. There were no statistically significant differences found in this study. An advantage of this study was that due to its methodology there were few missing data, which thus avoided certain biases. Another strength was that it was a large-scale study quantifying the effect of lifestyle effect factors on the risk of developing chronic physical health conditions amongst autistic adults. Some limitations of this study included a low statistical power due to the sample size, which did not yield a reliable effect size difference between autistic and non-autistic adults, as well as rare medical conditions. There was also the possibility of certain biases, since the participants provided self-report survey measures. Sampling bias was also possible, since the study was advertised on various autism social support groups and social media sources. Another limitation was that individuals who were unable to complete the online self-report survey were excluded. This confined the data to people with internet access, as well as a specific level of intellectual capacity and physical competence, which is unlikely to reflect the whole autistic community. Another drawback was that because the researchers excluded everyone with a self-diagnosis of autism or suspected autism, the control sample may not be typical of the broader population. Lastly, autistic males were highly under sampled, which could have affected the results and limited the statistical power of the study [42].

## 4. Discussion

This systematic review aims to summarize QoL and co-occurring medical conditions amongst individuals with ASD, with a special focus on autistic females.

### 4.1. QoL

The three QoL studies included in this systematic review showed that individuals with autism tend to have a lower QoL compared to neurotypical individuals. Women with a primary ADHD diagnosis tend to have greater proportions of reported chronic pain compared to women with a primary ASD diagnosis. The findings of chronic pain in women with ASD or ADHD (76.6%) were substantially greater than in the 2012 Canadian Community Health Survey (28.0%). Moreover, most adults with ASD were found to be negatively affected by at least one GI-related symptom. Other problems related to sleep and social support were also found to affect QoL particularly in children. Prior research showed an unfavorable relationship between sleep-associated problems, physical HRQoL, and psychosocial HRQoL in children with ASD [44]. The third QoL study showed that sleep disturbances in early childhood can persist into adulthood for individuals with ASD, and thus these effects can remain and negatively affect the QoL in adulthood. This study also found that lower social functioning had negative relationships with QoL across various dimensions, suggesting that how a person interacts with their environment has a significant impact on how they experience life. According to Khanna et al. [45], individuals with a physical illness diagnosis had lower physical QoL compared to individuals without this diagnosis. Furthermore, the QoL amongst individuals with ASD could indicate some differences between males and females. According to one of these research studies, females with ASD had a higher QoL than males with ASD based on the social domain of the World Health Organization Quality of Life (WHOQOL-BREF), implying that females with ASD are more socially oriented and capable of maintaining more friendships [8]. Another study showed that as females with ASD increase in age, their mental HRQoL improves, whereas this was not observed in males. Thus, this could mean that older women with ASD may have a higher capacity of mental health resilience. To help improve social support in women with ASD, one of the studies found that relaxation education interventions could help improve the HRQoL in women with ASD. It is evident that men with ASD and women with ASD are not similarly affected by reductions in HRQoL. 

### 4.2. Dementia 

Another common comorbidity in individuals with ASD is the prevalence of psychiatric conditions such as dementia. The findings show that those who had both ASD and a dementia-related condition designated as a cause of death were considerably less likely to die from a dementia-related disorder. This is consistent with previous research on age-related cognitive performance reductions in people with autism [46,47,48]. Furthermore, males are much more likely than females to have a dementia-related illness. Individuals with ASD do survive into their elderly years and thus need supportive services, as ASD is a lifelong disorder [49].

### 4.3. Epilepsy

Epilepsy is associated with significant burdens in those who are affected, reducing quality of life and everyday functioning [50,51]. When looking at ASD identification, there are several distinct attributes that are different, which may help to explain why females need to present with more severe symptoms than men in order to receive an ASD diagnosis [12]. Additionally, this has been linked to a higher incidence of seizures [52]. The likelihood of developing seizures may also be increased in females with ASD due to a larger burden of mutations [53]. Finally, it was not shown that women with intellectual disabilities were more likely to have epilepsy. These results on the occurrence and extent of epilepsy in adults with autism demonstrate the need for incident epilepsy screening and treatment, particularly in women with autism with intellectual impairment [50].

### 4.4. Post-Traumatic Stress Disorder

There has been little empirical data regarding the co-occurrence of ASD and PTSD. The findings of a study on PTSD and ASD indicate that there is a significantly high number of adults with ASD and with Post Traumatic Stress Syndrome (PTSS) [20]. In particular, females with autism are more likely to get PTSS, since they are at a higher risk of being exposed to traumatic events in their life [28]. Experiences in social spheres were shown to be potentially more traumatic, particularly for females diagnosed with autism. These females also reported significantly higher symptoms of hyper-arousal than males with ASD. Furthermore, it is also likely that individuals with ASD received less social support than typical individuals [23]. Women with ASD were found to have a higher risk of death from suicide [9]. This is important because social support can play a major role during trauma encounters and potentially prevent suicide-related deaths [54].

### 4.5. Other Medical Conditions 

Individuals with ASD in Utah, USA, who were diagnosed with a higher severity of autism symptoms were observed to have a higher prevalence of other medical conditions, hospitalizations, and surgeries [29]. These comorbidities were higher in females and obese individuals [29]. Furthermore, the level of intellectual disability was not shown to be associated with other comorbid medical disorders, even though it was shown to appear in neurodevelopmental traumas affecting specific organ systems [29]. These comorbidities highlight the importance of these individuals receiving attenuative medical care and indicate that the medical conditions experienced in childhood do extend into adulthood. 

### 4.6. Depression

Depression is common among adults with ASD, but it is unclear how frequently these individuals have access to treatment. Studies have observed that there is a significantly elevated prevalence of depression in autistic young adults (47%) and lifetime rated depression (65%). Some factors that possibly contributed to these results could be the effect of adults already experiencing depression, since the participants were familiar with the aims of the study. Another factor could be that individuals with ASD with a lower severity of autism with higher cognitive functions have been found to be more likely to experience depression [55,56,57]. The gender differences in depression based on these findings may not be due to sex differences in the base rates of elevated depression symptoms. Instead, they could be due to the reflection of sex differences in the identification of depression.

Prior studies have found high levels of depression and related anxiety in adults with ASD [58]. In particular, females with autism have been found to have increased amounts of anxiety during their young to middle adulthood. A number of studies have suggested that females with ASD have self-reported feelings of anxiety-like symptoms. These findings suggests that techniques to alleviate depression and related anxiety should be put into clinical practice for females diagnosed with ASD.

### 4.7. Eating Disorders

Males and females diagnosed with ASD both have difficulty adapting to their eating behaviors. They also having difficulty performing two tasks at the same time while eating. Furthermore, it is difficult for males and females with ASD to adapt and be social during their meals. They prefer to eat alone and are less socially inclined while eating. Specifically, women with ASD showed more eating-related problems on most subscales, excluding Pica and Motor control. The results of this study show that eating problems in children with ASD are still present in their adulthood, particularly in females, to the point where they affect day-to-day life. Thus, this finding potentially indicates that girls with ASD experiencing eating problems have an increased susceptibility to developing eating disorders later in life. Women with ASD were also found to have an increased number of sensory-related issues compared to men with ASD [7].

### 4.8. Limitations

A general lack of well-designed investigations is one of the research’s shortcomings when it comes to evaluating the prevalence of neurosensory disorders and medical comorbidities in people with ASD. The absence of a suitable control group is one of the most crucial restrictions to take into account. Because of this, it is challenging to draw reliable conclusions regarding people with ASD, particularly women, and their associated comorbidities. The other common limitation is due to small sample sizes. The inclusion of a small cohort makes it more difficult to generalize the conclusions to a wider ASD population. Furthermore, the majority of the research utilized subjective sampling, with participants assessing themselves. This can lead to biases, since the study is based on the replies of participants rather than an observation or quantitative approach produced by researchers. Additionally, an important limitation to consider is that a large amount of these studies did not validate the assessed diagnoses via a standardized clinical exam. Many of these diagnoses were based on the original diagnosis provided by the first practitioner. Furthermore, several of these studies included people with ASD who had ordinary or above-average cognitive ability. This also makes it more difficult to generalize to ASD groups with below-average cognitive ability. Finally, several of these studies failed to account for the drugs that these subjects were taking, which might have influenced the findings. Future research with a large sample size and an adequate control group is needed to better understand the incidence of neurosensory disorders and medical comorbidities in people with ASD. A greater understanding of the underlying comorbidities in the ASD community would aid in the development of focused therapies and support, particularly for autistic females, in the quest of enhancing the quality of life of afflicted persons and their families.

## 5. Conclusions

In individuals with ASD, our systematic review paper highlights decreased QoL and the incidence of neurosensory abnormalities, as well as medical comorbidities such as dementia, PTSD, anxiety, and depression. Based on these research studies, females diagnosed with ASD present with varying types of pain, frequent headaches, poor life events, and more medical comorbidities. Compared with men diagnosed with ASD, autistic women are more likely to have more CSS diagnoses, a higher mean age death, higher prevalence of epilepsy and formal depression diagnoses, higher anxiety-like symptoms, and higher levels of stress. An interesting finding was that older age in women with ASD was demonstrated to improve mental HRQoL. As these women become older, they learn and adapt methods to better manage the autism they are diagnosed with. With the progress of technology and artificial intelligence, ASD may be detectable at a younger age [59]. Early detection of ASD-like symptoms through technology and identification of specific comorbidity traits would be beneficial so that these individuals can learn how to manage ASD [60,61]. Furthermore, some of the studies considered in the present systematic review either included male participants or did not report gender-specific results. Future studies are needed to analyze female participants and compare profiles with male counterparts in order to better understand gender disparities. The findings of these research will assist in the development of successful specialized support systems for the population with ASD, particularly females with autism.

## Data Availability

Not applicable.
